# Supraventricular tachycardias in the first year of life: what is the best pharmacological treatment? 24 years of experience in a single centre

**DOI:** 10.1186/s12872-020-01843-0

**Published:** 2021-03-15

**Authors:** Guglielmo Capponi, Gilda Belli, Mattia Giovannini, Giulia Remaschi, Alice Brambilla, Francesca Vannuccini, Silvia Favilli, Giulio Porcedda, Luciano De Simone

**Affiliations:** 1 Department of Health Sciences, Post-Graduate School of Paediatrics, Anna Meyer Children’s University Hospital, Florence, Italy; 2 Neonatology Department and Neonatal Intensive Care Unit, Careggi University Hospital, Florence, Italy; 3 Cardiology Unit, Anna Meyer Children’s University Hospital, Viale Gaetano Pieraccini 24, 50139 Florence, Italy

**Keywords:** Supraventricular tachycardia, Infant, Flecainide, Beta-blockers

## Abstract

**Background:**

Supraventricular tachycardias (SVTs) are common in the first year of life and may be life-threatening. Acute cardioversion is usually effective, with both pharmacological and non-pharmacological procedures. However, as yet no international consensus exists concerning the best drug required for a stable conversion to sinus rhythm (maintenance treatment). Our study intends to describe the experience of a single centre with maintenance drug treatment of both re-entry and automatic SVTs in the first year of life.

**Methods:**

From March 1995 to April 2019, 55 patients under one year of age with SVT were observed in our Centre. The SVTs were divided into two groups: 45 re-entry and 10 automatic tachycardias. As regards maintenance therapy, in re-entry tachycardias, we chose to start with oral flecainide and in case of relapses switched to combined treatment with beta-blockers or digoxin. In automatic tachycardias we first administered a beta-blocker, later combined with flecainide or amiodarone when ineffective.

**Results:**

The patients’ median follow-up time was 35 months. In re-entry tachycardias, flecainide was effective as monotherapy in 23/45 patients (51.1%) and in 20/45 patients (44.4%) in combination with nadolol, sotalol or digoxin (overall 95.5%). In automatic tachycardias, a beta-blocker alone was effective in 3/10 patients (30.0%), however, the best results were obtained when combined with flecainide: overall 9/10 (90%).

**Conclusions:**

In this retrospective study on pharmacological treatment of SVTs under 1 year of age the combination of flecainide and beta-blockers was highly effective in long-term maintenance of sinus rhythm in both re-entry and automatic tachycardias.

## Background

Supraventricular tachycardias (SVTs) are the most common arrhythmia occurring in the first year of life, with an incidence of 1/250–1/1000 live births and 1/10 in patients with congenital heart diseases [1]. In these patients, the aetiology of SVT must be sought in immaturity of the conduction system and a major sensitivity to catecholamines [2, 3]. In recent years the management of the prevention of supraventricular tachycardia recurrences has shifted towards the use of Class III antiarrhythmic drugs (sotalol and amiodarone) or Class IC drugs (flecainide and propafenone), with success rates comparable with digoxin and beta-blocking agents. However, the “gold standard” therapy in terms of safety and efficacy for long-term treatment has not been established [4]. Our retrospective study evaluated the different antiarrhythmic response of re-entry and automatic tachycardias to maintenance therapy mainly with Class IC drugs and beta-blockers, used either alone or in combination.

## Methods

We performed an observational retrospective study among patients suffering from SVTs occurring in the first year of life and referred to the Cardiology Unit of the Anna Meyer Children’s University Hospital in Florence from March 1995 to April 2019. All the patients were treated in the Intensive Care Unit and data were collected by selecting and reviewing paper-based medical records, and since January 2000, electronic medical records. The patients’ inclusion criteria were an SVT at onset between the fetal period and the end of the first year of life. We divided the patients into two sub-groups: re-entry or automatic tachycardias. Since they have the same mechanism, we included atrioventricular re-entry tachycardias (AVRT), atrioventricular nodal re-entry tachycardias (AVNRT), and paroxysmal junctional reciprocating tachycardias (PJRT) in the re-entry tachycardias. These different arrythmias were diagnosed on the basis of 12-lead ECGs (trans-thoracic or trans-oesophageal) according to the European Society of Cardiology practice guidelines and other criteria [5–7]: (a) in an AVRT, the P wave is usually identifiable in the ascending or descending branch of the T wave, the PR is longer than the RP^’^ interval, the T wave follows about 70 ms after the QRS; (b) in an AVNRT, the P wave is identifiable as a pseudo r1 wave in V1 and a pseudo s wave in inferior leads; (c) a PJRT is characterized by an R–P’ interval longer than P–R with the P–R interval being normal or short, negative P waves in leads II, III and aVF and the absence of “warm up” at SVT onset; (d) automatic tachycardia is recognized by a progressive shortening in the P–P interval at the onset of SVT and an R–P’ interval longer than the P–R interval.

We also used intravenous adenosine as an additional diagnostic test: in re-entry forms it abruptly restores the sinus rhythm, whereas it only reduces the ventricular rate in most automatic forms [8]. All the patients were treated with acute therapy, defined as treatments used with the aim of restoring the sinus rhythm: adenosine, intravenous or oral flecainide, intravenous amiodarone or intravenous propafenone, and non-pharmacological treatments (diving reflex, trans-oesophageal stimulation or direct current shock). We used flecainide as a first-line drug in re-entries, whereas we decided to start with a beta-blocker in the automatic forms [9, 10]. In case of unresponsiveness, other drugs were added. The short and long-term efficacy of flecainide and beta-blockers was assessed. Flecainide was administered orally at a dose ranging from 50 to 120 mg/m^2^/day and when the sinus rhythm was not stable for more than 3 half-lives, we decided to add a beta-blocker (sotalol 0.5–2.5 mg/kg/day, propranolol 1–3 mg/kg/day, nadolol 1–5 mg/kg/day) or digoxin 0.005–0.0075 mg/kg/day. We used amiodarone, 75–250 mg/m^2^/day, as a third-line agent [4, 11, 12]. Digoxin has been replaced by beta-blockers since 2000. In automatic tachycardias we decided to start with beta blockers (propranolol, nadolol or sotalol) at the above dosage schedule. When a stable sinus rhythm was not achieved after 3 half-lives, we decided to add flecainide and, in case of unresponsiveness, shifted to the combination with amiodarone. Since October 2014 the serum concentration of flecainide has been assessed in all patients by liquid chromatography tandem-mass spectrometry and it has been kept in a range of 200–800 ng/ml. Measuring the drug concentration helped us to identify under or overdosage and allowed for changing the oral therapy, irrespective of clinical symptoms or ECG alterations. We also reported the time to sustained sinus rhythm, defined as the days between the first administration of an antiarrhythmic agent and achievement of the stable sinus rhythm. After discharge, each patient had home monitoring for 1 year at the most, and they all underwent a regular follow-up in our Cardiology Unit where we documented any supraventricular tachycardia relapses, therapeutic variations or adverse effects. In the absence of recurrences during the previous 6 months or after the first year of life with at least one week without antiarrhythmic therapy, we performed a trans-oesophageal study. When we were not able to induce a re-entry arrhythmia, the drug therapy was discontinued. The statistical analysis can be divided into two parts: for one we used descriptive values as mean, standard deviation (SD), median and discrete values as percentage using Excel algorithms; for the other we used the t-test to compare time to cardioversion in re-entry and automatic tachycardias.

## Results

### Study population

From March 1995 to April 2019, 55 patients with SVTs occurring in the first year of life were monitored at our centre and satisfied our criteria. 6/55 patients (10.9%) had congenital heart disease (CHD): 3 ventricular septal defects, 1 ventricular septal defect associated with atrial septal defect, 1 non-compacted left ventricular cardiomyopathy associated with ventricular septal defect and 1 Ebstein anomaly. In 7/55 patients (12.7%), the arrhythmia occurred during fetal life. Median postnatal age at onset was 12.5 days (1–303 days, mean 40.9 days ± 77.6 days SD). Of these, 45/55 (81.2%) had a re-entry tachycardia (Table 1): 40/45 (88.8%) had AVRT, 4/45 (8.8%) AVNRT, and 1/45 (2.2%) a PJRT. The patients’ heart rate ranged between 200 and 340 bpm. The clinical symptoms at onset were documented in 43/45 patients (95.5%): 2/43 (4.6%) had cardiogenic shock, 19/43 (44.1%) symptoms related to heart failure (dyspnoea, failure to thrive, pallor, drowsiness), and 22/43 (51.1%) minor symptoms (restlessness, persistent crying). In 10/55 patients (18.1%), the ECG detected automatic tachycardias (Table 2); heart rate ranged between 180 and 300 bpm. Clinical presentation was cardiogenic shock in 1/10 patients (10.0%), heart failure-related symptoms in 2/10 (20.0%), and minor symptoms in 7/10 (70.0%).

**Table 1:** Characteristics of the patients with re-entry tachycardias

	Types of re-entry	Onset (days)	CHD	Maximum HR (bpm)	Symptoms	Maintenance therapy	Time to CV (days)
1	AVRT	32		300	Minor	Flecainide + nadolol	7
2	AVRT	Fetal		280	Minor	Flecainide + nadolol	15
3	AVRT	1		280	Minor	Flecainide + sotalol	8
4	AVRT	64		240	Minor	Flecainide	1
5	AVRT	18		300	HF	Flecainide	1
6	AVNRT	1		240	Minor	Flecainide	1
7	AVRT	230		300	HF	Flecainide	1
8	AVRT	2		270	Minor	Flecainide	1
9	AVRT	12		250	Minor	Flecainide	1
10	AVRT	14			Minor	Flecainide	1
11	AVRT	10		280	HF	Flecainide	1
12	AVRT	13		320	Shock	Flecainide	1
13	AVRT	1		250	HF	Amiodarone + nadolol	22
14	AVRT	1		320	Minor	Flecainide	1
15	AVRT	256		300	Minor	Flecainide + sotalol	7
16	AVRT	56		260	HF	Flecainide	1
17	AVNRT	6		260	Minor	Flecainide	1
18	AVRT	1		261	Minor	Flecainide + propranolol	15
19	AVNRT	13	VSD	215	Minor	Flecainide	1
20	AVRT	15			HF	Flecainide + digoxin	7
21	AVRT	26		280		Flecainide + digoxin	4
22	AVRT	11				Flecainide + digoxin	2
23	AVRT	Fetal			HF	Flecainide + sotalol	15
24	AVRT	10		340	HF	Flecainide	1
25	AVRT	36		280	Minor	Flecainide + nadolol	3
26	AVRT	72		300	HF	Flecainide	1
27	AVRT	27		280	Minor	Flecainide + digoxin	4
28	AVRT	8		300	Shock	Flecainide	D
29	AVRT	15		260	HF	Flecainide	1
30	AVRT	Fetal		300	HF	Flecainide + digoxin	4
31	AVRT	15	Ebstein	200	HF	Flecainide + digoxin	3
32	AVRT	3		300	Minor	Flecainide	1
33	AVRT	Fetal		220	HF	Flecainide	1
34	AVRT	12	ASD + VSD	260	HF	Flecainide + propranolol	7
35	AVRT	1		240	Minor	Flecainide	1
36	AVNRT	12		220	HF	Flecainide	1
37	AVRT	224		280	Minor	Flecainide + nadolol	6
38	AVRT	21		201	HF	Flecainide	1
39	AVRT	24		300	HF	Flecainide + nadolol	5
40	AVRT	7		300	Minor	Flecainide + nadolol	7
41	AVRT	1		280	Minor	Flecainide	1
42	AVRT	36		290	HF	Amiodarone + nadolol	15
43	AVRT	11		280	Minor	Flecainide + nadolol	7
44	AVRT	1		225	Minor	Flecainide	1
45	PJRT	Fetal		217	HF	Flecainide + nadolol	15

*AVRT* atrioventricular re-entry tachycardia,* CV* cardioversion, *AVNRT* atrioventricular nodal re-entry tachycardia, *PJRT* paroxysmal junctional re-entry tachycardia, *ASD* atrial septal defect, *CHD* congenital heart diseases, *D* death, *Ebstein* Ebstein’s anomaly, *HF* heart failure, *HR* heart rates, *minor* minor symptoms, *NCLV* non-compacted left ventricular cardiomyopathy, *shock* cardiogenic shock, *VSD* ventricular septal defect

**Table 2:** Characteristics of the patients with automatic tachycardias

	Onset (days)	CHD	Maximum HR (bpm)	Symptoms	Maintenance therapy	Time to CV (days)
1	Fetal		240	Minor	Flecainide + nadolol	40
2	Fetal		220	Minor	Flecainide + sotalol	22
3	285		280	Minor	Flecainide + sotalol	20
4	2	VSD + NCLV	210	Minor	Amiodarone + nadolol	40
5	303		300	Shock	Flecainide + nadolol	23
6	36	VSD	180	HF	Flecainide + nadolol	22
7	1		240	HF	Flecainide + sotalol	32
8	13		230	Minor	Nadolol	6
9	1		195	Minor	Propranolol	26
10	6	VSD	250	Minor	Sotalol	10

*CHD* congenital heart diseases,* CV* cardioversion, *HF* heart failure, *HR* heart rates, *minor* minor symptoms, *NCLV* non-compacted left ventricular, *VSD* ventricular septal defect

### Responses

**Fig. 1:**
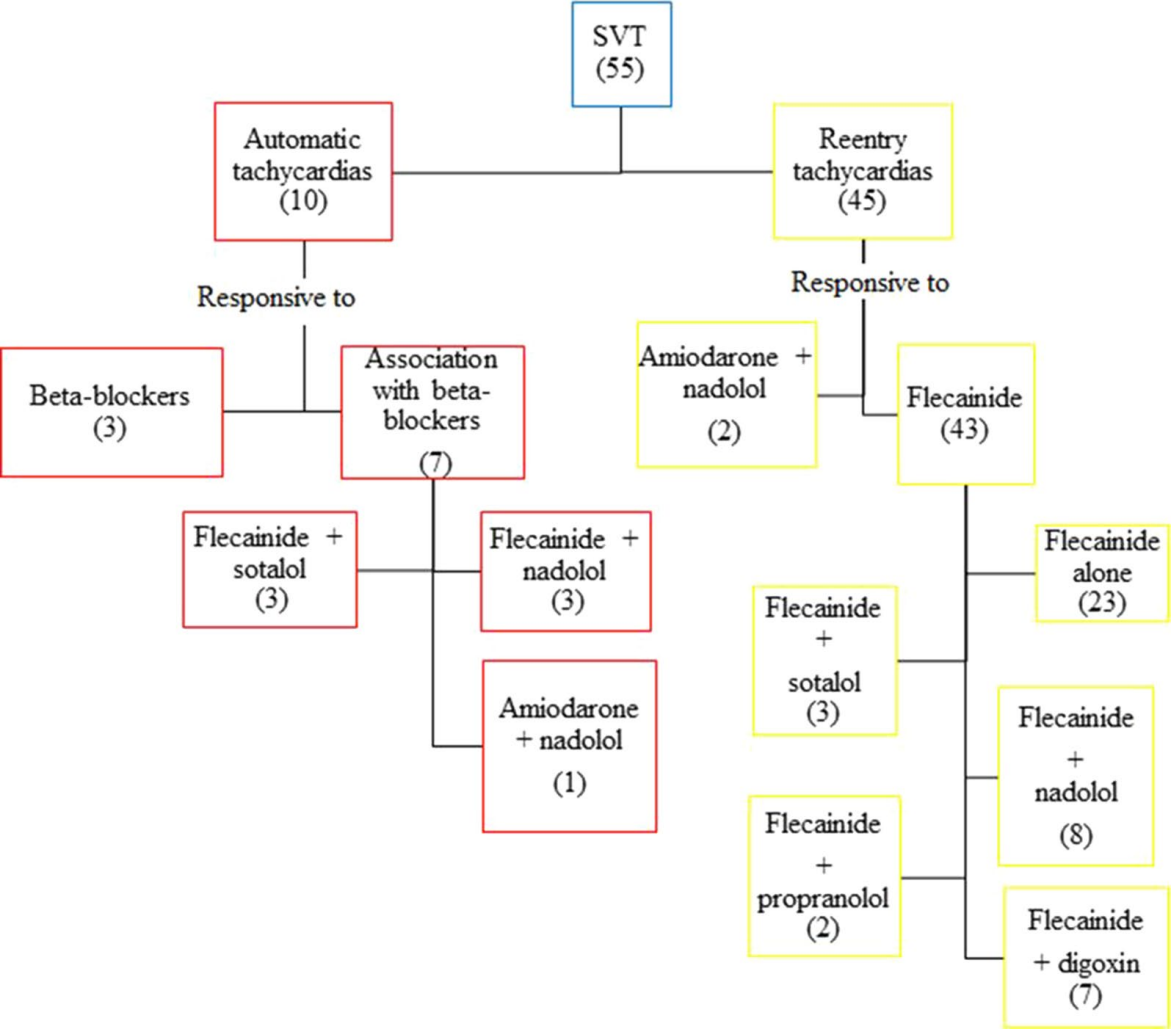
Flow chart of responses

During the median 35 months’ follow-up (from 0 to 289 months), we obtained different responses in re-entry and automatic tachycardias (Fig. 1). In re-entry tachycardias, flecainide was effective as monotherapy in 23/45 patients (51.1%), while in 20/45 (44.4%), a stable cardioversion was achieved after adding digoxin in 7/20 patients (35.0%), sotalol in 3/20 (15.0%), nadolol in 8/20 (40.0%), and propranolol in 2/20 (10.0%). For 2/45 patients (4.4%), flecainide was ineffective: in both cases the amiodarone plus nadolol combination was effective. As regards automatic tachycardias, the beta-blockers nadolol, propranolol and sotalol in monotherapy were effective in 3/10 patients (30.0%). The others, (7/10) (70.0%), required the combination of beta-blockers with another drug: flecainide in 6/7 (85.7%) cases, and amiodarone in 1/7 (14.2%). In the majority of patients, flecainide was effective with two or three administrations per day, in 2/52 cases (3.8%), however, the number of doses had to be increased: one patient was given six doses of flecainide and four of nadolol a day and the other patient was stabilized with six doses of flecainide and three of sotalol a day. Unlike re-entry supraventricular tachycardias, stable cardioversion was delayed in automatic tachycardias. In re-entry tachycardias, we obtained sustained sinus rhythm between 1 and 22 days, while in automatic tachycardias we achieved the same result between 6 and 40 days (*p* < 0.05) (Table 3). In all but one case, 1/55 (QTc equal to 500 ms), the QTc interval was in the normal range. At the last follow-up (median 35 months), 29/55 (52.7%) patients were still in maintenance therapy, while 19/55 (34.5%) had stopped their drug therapy in accordance with the trans-oesophageal study. 5/55 (9.0%) patients underwent radiofrequency ablation at 7, 10, 12 (2 patients) and 13 years of age. Maintenance therapy had been carried out before discontinuation for a median of 16 months (from 10 to 72 months). We had six hospital readmissions: three patients received flecainide and nadolol, two flecainide and sotalol, and one propafenone; stable maintenance of sinus rhythm was obtained thanks to dose increments and in one patient by shifting to amiodarone and nadolol.

**Table 3:** Time to cardioversion (days)

	Re-entry tachycardias	Automatic tachycardias	
Mean ± SD (days)	4.5 ± 5.2	24.1 ± 11.1	*p* < 0.05
Median (days)	1 (1 to 22)	22.5 (6 to 40)	

### Safety

One patient died during follow-up because of decompensated heart failure related to glycogenosis type 2. Flecainide caused significant adverse events in 2/52 (3.8%) patients: one had severe bradycardia (80 rpm) with wide QRS and atrioventricular dissociation, transitory pallor, and hypotonia, followed by rapid and complete recovery after discontinuing treatment. Another death involved an outpatient unsuccessfully treated with intravenous flecainide elsewhere, who, after administration of the correct oral dose of flecainide, experienced severe intraventricular conduction disorders, bradycardia, ventricular arrhythmias, evolving into asystole and cardiac arrest, unresponsive to cardiopulmonary resuscitation treatment. Both adverse events were related to accidental overdosage: in the first, the flecainide concentration was 2 times higher, and in the second, 6 times higher than normal. Two other patients experienced minor complications: psychomotor agitation and QT prolongation, without causing major arrhythmias. Beta-blocker therapy was fully tolerated in all patients.

## Discussion

Many anti-arrhythmic drugs are used in both acute and maintenance treatment of re-entry and automatic tachycardias in the first year of life; however, to date no consensus exists regarding the most effective drug, and there is still a wide variability in the treatments. According to the European guidelines, in case of re-entry tachycardias the first antiarrhythmic choice should be a Class 1A, Class 1C or Class 3 antiarrhythmic agent, except for amiodarone that should only be used if other drugs fail to control the arrhythmia. In case of automatic tachycardias, the first choice should be digoxin, with a Class 1C agent or beta-blocker added in case of failure, and using amiodarone as a third-line drug [4]. Our data demonstrate the effectiveness of flecainide and beta-blockers for a long-term follow up, starting from neonatal age up to childhood. In re-entry tachycardias we focused on the antiarrhythmic properties of oral flecainide as maintenance therapy. Flecainide, an IC anti-arrhythmic drug, has optimal pharmacodynamic properties, namely, the ability to slow the rate of diastolic depolarization plus it is highly effective in inducing anterograde and retrograde conduction block on accessory pathways. It also presents good pharmacokinetic properties, including relatively fast oral absorption and a long half-life elimination of about 11–12 h [13, 14]. Thanks to these properties, this drug showed high efficacy in 73–100% of supraventricular tachycardias [15]. Unlike in adulthood, where a significant incidence of pro-arrhythmic effects was reported in the CAST study, flecainide-induced arrhythmias were documented in less than 7% of the paediatric population [15, 16]. Propafenone, another IC anti-arrhythmic drug, demonstrated good results in clinical trials [17]. In a prospective study comparing four anti-arrhythmic drugs in long-term prophylaxis of SVT, it appeared to be less effective than flecainide [10]. Few studies are reported with sotalol, a Class III anti-arrhythmic agent with beta-blocking properties; although high effectiveness has been reported with elevated doses in paediatric SVTs, we decided to use it in combination with flecainide, given the encouraging results of this combination [18]. Beta-blockers in monotherapy might be an alternative choice, both in re-entry and automatic tachycardias. In the SAMIS trial, one of the first prospective studies in infants, long-term treatment with propranolol in monotherapy demonstrated non-inferiority when compared with digoxin in monotherapy [19]. In automatic atrial tachycardia, beta-blockers were used in monotherapy or combined mostly with digoxin or flecainide [20]. Finally, amiodarone, a third-class anti-arrhythmic agent, can only be considered an alternative for infants with resistant supraventricular tachycardias due to its well-known adverse events: in a recent cohort of 150 paediatric patients receiving amiodarone in acute and long-term treatment, 50.8% developed thyroid dysfunction, especially the newborns (66.7%) [12]. Furthermore, a recent comparative study between flecainide and amiodarone for the treatment of paediatric supraventricular tachycardias demonstrated the non-inferiority of the former, both in patients with congenital heart disease and those with structurally normal hearts. Lastly, in all the patients in whom amiodarone was ineffective, the shift to flecainide allowed for controlling the arrhythmia [11]. Perry et al. [14] observed 45 patients with supraventricular tachycardias (both re-entries and automatics) and showed that flecainide controlled about 81% of cases. O’Sullivan et al. [21] reported that 39 infants with sustained atrioventricular re-entry tachycardias non-responsive to digoxin were treated with flecainide, with about 96% of success. Ferlini et al. [9] showed that neonates could be treated with this drug both in acute and mid-term therapy with an effectiveness of approximately 85%. Our data showed an efficacy of about 51.1% in re-entry tachycardias as monotherapy; however, when we combined it with beta-blockers or digoxin, a stable cardioversion was achieved in up to 95.5% of cases. Digoxin has been reported to be successful when combined with flecainide both in fetal and neonatal supraventricular tachycardias [20, 22]. The effectiveness of combined treatment compared to monotherapy may be related to the enhanced adrenergic tone of the neonate, representing a trigger for re-entry arrhythmias**.** In order to achieve stable rhythm control, automatic tachycardias, known to be more resistant to incessant antiarrhythmic treatment, require a pharmacological combination more frequently than re-entry forms [20].

Price et al. [23] considered 10 patients with supraventricular tachycardias (eight re-entry and two automatic) in the first year of life who were unresponsive to at least two anti-arrhythmic agents: the combination of flecainide and sotalol was effective in all patients in an average of twelve days. Also in our experience, although sotalol is scarcely effective in monotherapy, when combined with flecainide, even at beta-blocking doses, it was able to obtain a stable cardioversion in resistant forms without prolonging the QTc interval [24]. Von Alvensleben et al. [25] considered 28 patients with supraventricular tachycardias (27 with re-entries and 1 with focal atrial tachycardia) under 2 years of age; nadolol alone resolved arrhythmia in 20/28 (71.4%) cases, and for the other six this result was achieved when combined with flecainide. Perry et al. [14] obtained stable cardioversion with flecainide alone for 9/13 (69.2%) patients with ectopic atrial tachycardias. In our study, beta-blockers proved to be effective in monotherapy in a minority (30%), but again, the combination with flecainide allowed for controlling almost all resistant tachycardias. Therefore, a substantial number of infants with re-entry supraventricular tachycardias and the majority with automatic forms, required the combination of two antiarrhythmic agents for a stable cardioversion. Flecainide and nadolol or sotalol seemed to be the most effective choice. The time to sustained sinus rhythm was significantly longer in automatic tachycardias than in re-entry forms [20, 26]. In one case, after obtaining a satisfactory rate control, we discharged the patient, and in the end observed stable conversion to sinus rhythm in all patients. In two cases a stable cardioversion was obtained when anti-arrhythmic drugs were administered more frequently than two or three times a day, with the strict recommendation to monitor ECG and blood flecainide concentration continuously in order to prevent toxicity [5, 27]. This different administration rate may be related to the variability of the drug’s pharmacokinetics, often reported in infants [14, 15]. Measuring flecainide blood concentration helped us to quantify the correct doses of the drug. Therefore, we strongly recommend this approach in clinical practice when possible in order to avoid life-threatening complications due to overdosage.

**Fig. 2:**
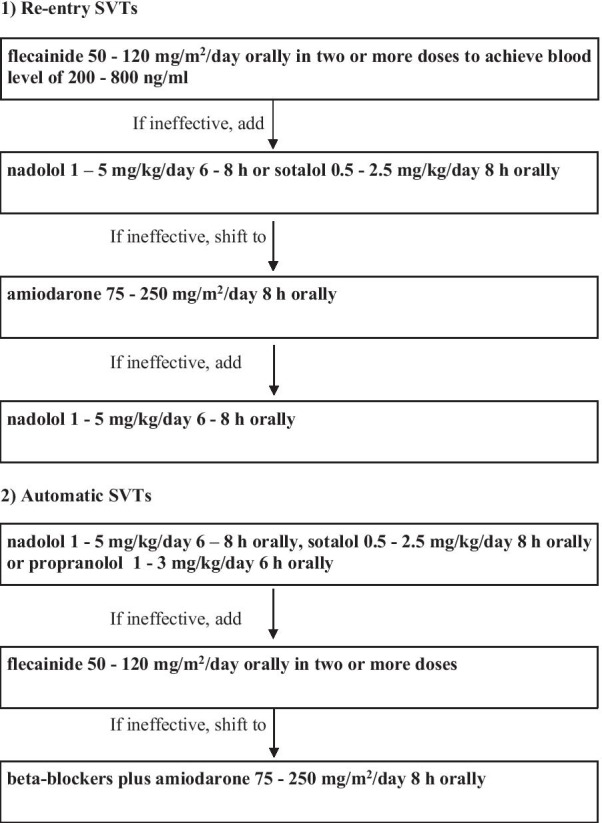
Protocol proposal for the treatment of supraventricular tachycardias in the first year of life

In line with our results, we have proposed a protocol for the treatment of infant supraventricular tachycardias, differentiating re-entry from automatic atrial tachycardias (Fig. 2). With regard to beta-blockers we decided to prefer nadolol over propranolol thanks to its longer half-life, and because of the excellent clinical results recently reported in maintenance therapy of SVT [25].

With flecainide treatment we observed two major ventricular arrhythmias. As for the patient coming from another hospital who died, flecainide toxicity could indeed have been the cause of death, possibly owing to a poor metabolizing mechanism, as also potentially reported in a recent Japanese study [28]. Incessant supraventricular tachyarrhythmias, ventricular tachycardias and severe bradycardias have been reported during flecainide treatment, therefore it is recommended to start therapy during hospitalization and to closely monitor the ECG and plasma concentration [15, 29]. Pro-arrhythmic effects and cardiac arrest seem to be more common among patients with underlying heart diseases or impaired systemic ventricular function than in those with a normal heart [30]. However, in a recent study, a comparison between the use of flecainide and other drugs in patients with cardiomyopathy or structural heart diseases showed that there were no differences in the incidence of cardiac arrest or death [31].

In our work, the gestational age at birth, birth weight and perinatal age were not predisposing factors influencing a different antiarrhythmic approach. Moreover, the efficacy of antiarrhythmic agents was not influenced by the presence of CHD defects, most of which were well-tolerated.

## Conclusions

Our experience shows that in re-entry SVTs, flecainide may be an effective treatment option as monotherapy; nonetheless, better results can be achieved in combination with beta-blockers. In automatic tachycardias, the association of flecainide with beta-blockers (nadolol or sotalol) seems highly effective in achieving heart rate control therapy with the aim of delayed cardioversion. In specific cases, tailored management could be required in order to maintain stable flecainide blood levels and obtain better control of arrhythmia, i.e. by increasing the number of administrations per day. It is imperative to remember that this drug should always be used with caution due to the percentage of adverse events: the possibility of monitoring the blood levels of flecainide may be helpful for this purpose.

## Study limitations

The main limitation of this study was its retrospective form, due to several changes in the clinical management of the patients’ therapy which did not allow for planning a prospective study. Moreover, the relatively small cohort of patients attenuated the statistical significance of our findings. In addition, the delineation of shared protocols concerning acute and long-term anti-arrhythmic treatment is advocated. For this purpose, more data from randomized controlled trials are needed.

## Data Availability

The data generated by and used in the study is available from the corresponding author upon reasonable request.

## Abbreviations

AVRT: Atrioventricular re-entry tachycardia
AVNRT: Atrioventricular nodal re-entry tachycardia
CHD: Congenital heart disease
PJRT: Paroxysmal junctional reciprocating tachycardia
SVT: Supraventricular tachycardia
